# Supraventricular Tachycardia as an Initial Manifestation of Malignancy-Related Cardiac Invasion: A Case Report

**DOI:** 10.7759/cureus.94666

**Published:** 2025-10-15

**Authors:** Varshitha Tumkur Panduranga, Ammar Abdulfattah, Asher Gorantla, Victor Freitas De Souza, Louis Salciccioli, Samy I. McFarlane, Sabu John

**Affiliations:** 1 Internal Medicine, State University of New York Downstate Health Sciences University, Brooklyn, USA; 2 Cardiology, State University of New York Downstate Health Sciences University, Brooklyn, USA; 3 Cardiovascular Disease, Department of Internal Medicine, State University of New York Downstate Health Sciences University, Brooklyn, USA; 4 Medicine, State University of New York Downstate Health Sciences University, Brooklyn, USA

**Keywords:** atrial tachyarrhythmias, cardiac malignancy, pericardial infiltration, pulmonary adenocarcinoma, supra ventricular tachycardia, tumor invasion

## Abstract

Cardiac tumors are exceedingly rare, with metastatic lesions occurring more frequently than primary tumors. These tumors generally present with non-specific symptoms, reflecting tumor location and invasion of adjacent structures, and diagnosis is frequently incidental. A 66-year-old male smoker presented with hemoptysis and weight loss. Echocardiography revealed external invasion and compression of the left atrium by a left lung mass. His hospital course was complicated by recurrent supraventricular tachycardia (SVT). CT imaging showed a necrotic lung mass infiltrating the left atrium and pericardium, and biopsy confirmed invasive pulmonary adenocarcinoma. Atrial tachyarrhythmias via direct malignancy-related cardiac infiltration as an initial presentation of cancer are exceedingly rare and can significantly delay diagnosis and treatment due to vague symptoms. However, this entity can lead to life-threatening arrhythmia, underscoring the importance of early diagnosis and management. This case highlights the importance of considering malignancy-related cardiac invasion in patients with underlying malignancy with new-onset arrhythmias, as with our case with pulmonary adenocarcinoma and potentially life-threatening SVT through direct pericardial and atrial infiltration and involvement.

## Introduction

Atrial arrhythmias as the initial manifestation of a mediastinal tumor invading cardiac structures are exceedingly rare, with only a few cases described in the literature. When cardiac metastases present early in the course of malignancy, they may be overlooked due to nonspecific symptoms, delaying diagnosis and increasing the risk of serious arrhythmic complications. Supraventricular tachycardia (SVT) and other atrial tachyarrhythmias can occur when malignancies extend through the pulmonary veins into the left atrial myocardium. Proposed mechanisms include tumor infiltration of the myocardial sleeves of the pulmonary veins, creating zones of altered conduction, mechanical stretching of atrial tissue provoking focal tachycardia, and invasion of autonomic plexuses at the pulmonary vein-atrial junction [1]. These structural and electrophysiological alterations can facilitate micro-reentrant circuits, triggered automaticity, or both [1]. This case highlights the importance of considering malignancy-related cardiac invasion in patients with new-onset arrhythmias, as pulmonary adenocarcinoma may directly give rise to potentially life-threatening supraventricular tachycardia (SVT) through atrial involvement.

## Case presentation

A 66-year-old man presented to the emergency department (ED) with progressive hemoptysis, cough, worsening fatigue, and weight loss for over a month. Vital signs (Table 1) were blood pressure of 122/69 mmHg, heart rate of 70 beats per minute, respiratory rate of 10 per minute, oxygen saturation of 97%, and temperature of 37.2 °C.

**Table 1:** Vitals and laboratory results CO2: carbon dioxide; BUN: blood urea nitrogen; eGFR: estimated glomerular filtration rate; ALT (SGPT): alanine aminotransferase (serum glutamic pyruvic transaminase); AST (SGOT): aspartate aminotransferase (serum glutamic oxaloacetic transaminase); WBC: white blood cell; RBC: red blood cell; MCV: mean corpuscular volume; MCH: mean corpuscular hemoglobin; MCHC: mean corpuscular hemoglobin concentration; RDW: red cell distribution width; MPV: mean platelet volume; NRBC: nucleated red blood cell; aPTT: activated partial thromboplastin time; PT: prothrombin time; INR: international normalized ratio; T4: thyroxine.

Test	Reference Range	Value
Blood Pressure	120/80 mmHg	122/69 mmHg
Heart Rate	60 - 100 beats.min	70 beats/min
Respiratory Rate	12 - 20 breaths/min	10 /min
Oxygen Saturation	95 - 100%	97%
Temperature	36.4 - 37.2 °C	37.2 °C
Anion Gap	5 - 15 mEq/L	19 (H)
Sodium	136 - 146 mmol/L	142
Potassium	3.5 - 5.0 mmol/L	4.8
Chloride	98 - 106 mmol/L	100
CO2	24 - 31 mmol/L	23 (L)
BUN	8.0 - 23.0 mg/dL	23.0
Creatinine	0.70 - 1.20 mg/dL	0.99
Glucose	70 - 99 mg/dL	146 (H)
Calcium	8.8 - 10.2 mg/dL	9.2
Total Protein	6.4 - 8.3 g/dL	7.9
Albumin	3.3 - 6.1 g/dL	3.5
Magnesium	1.60 - 2.60 mg/dL	1.98
Phosphorus	2.5 - 4.5 mg/dL	4.4
eGFR (CKD-EPI 2021)	>=60.0 ml/min/1.73m2	>60.0
ALT (SGPT)	0 - 41 U/L	8
AST (SGOT)	10 - 50 U/L	20
Alkaline Phosphatase	35 - 145 U/L	61
Total Bilirubin	0.0 - 1.2 mg/dL	0.7
WBC	4.50 - 10.90 K/uL	9.49
RBC	4.20 - 6.10 M/uL	4.72
Hemoglobin	14.0 - 18.0 g/dL	13.5 (L)
Hematocrit	42.0 - 52.0 %	45.4
MCV	78.0 - 95.0 fL	96.2 (H)
MCH	26.6 - 31.6 pg	28.6
MCHC	30.5 - 35.5 g/dL	29.7 (L)
RDW	11.5 - 15.1 %	15.9 (H)
Platelets	130 - 400 K/uL	451 (H)
MPV	6.4 - 12.2 fL	9.7
Monocytes %	2.4 - 9.2 %	11.0 (H)
Monocytes Abs	0.09 - 1.11 K/uL	1.04
Neutrophils %	38.7 - 60.3 %	63.1 (H)
Neutrophils Abs	1.51 - 7.30 K/uL	5.99
Lymphocytes %	22.4 - 49.0 %	24.6
Lymphocytes Abs	0.88 - 5.93 K/uL	2.33
Eosinophils %	0.0 - 8.6 %	0.4
Eosinophils Abs	0.00 - 1.04 K/uL	0.04
Basophils %	0.0 - 1.0 %	0.6
Basophils Abs	0.00 - 1.20 K/uL	0.06
Immature Gran %	0.0 - 0.0 %	0.3 (H)
Immature Gran Abs	0.00 - 0.00 K/uL	0.03 (H)
NRBC %	<=0.0 %	0.0
NRBC Abs	<=0.00 K/uL	0.00
aPTT	25 - 37 Seconds	31
PT	9.4 - 12.5 Seconds	13.5 (H)
INR	0.8 - 1.2 Ratio	1.2
Thyroid Stimulating Hormone	0.270 - 4.200 uIU/mL	1.740
Free T4	0.93 - 1.70 ng/dL	1.19

**Figure 1:**
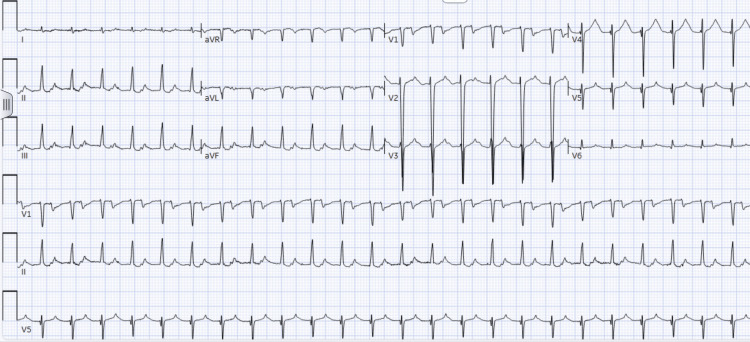
EKG showing SVT (likely AVNRT) SVT: supraventricular tachycardia; AVNRT: atrioventricular nodal reentrant tachycardia.

Physical examination was remarkable for decreased breath sounds on the right side of the chest. Chest X-ray (CXR) showed complete opacification of the left hemithorax with leftward shift of the mediastinum along with right-sided pleural effusion. Basic laboratory results, including troponin I and N-terminal pro-brain natriuretic peptide (NT-pro BNP), were negative. Electrolytes were within normal limits. The patient had no prior history of arrhythmia, smoking, or cardiac-related history. Initially, the electrocardiogram (EKG) (Figure 1) showed supraventricular tachycardia (SVT), likely atrioventricular nodal reentrant tachycardia (AVNRT), with a heart rate of 165 beats per minute.

**Figure 2:**
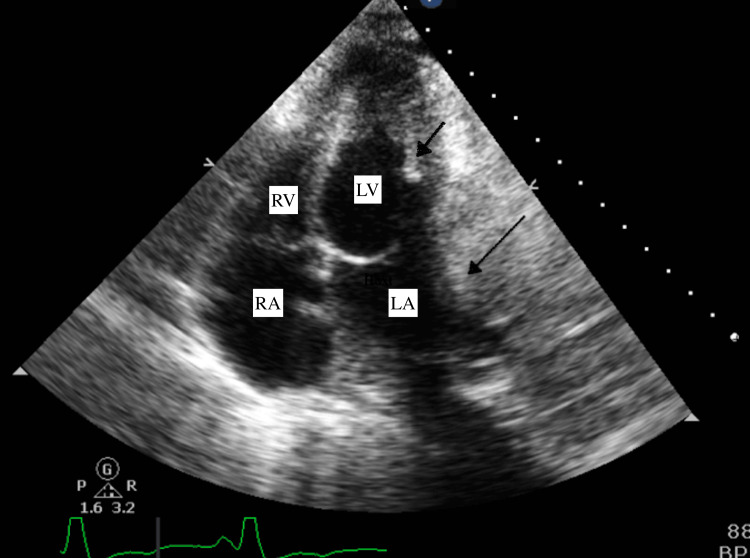
Echocardiographic apical 4 chamber view Showing the lung mass is pressing and invading the base of the left ventricle and left atrium (short and long black arrow respectively).

**Figure 3:**
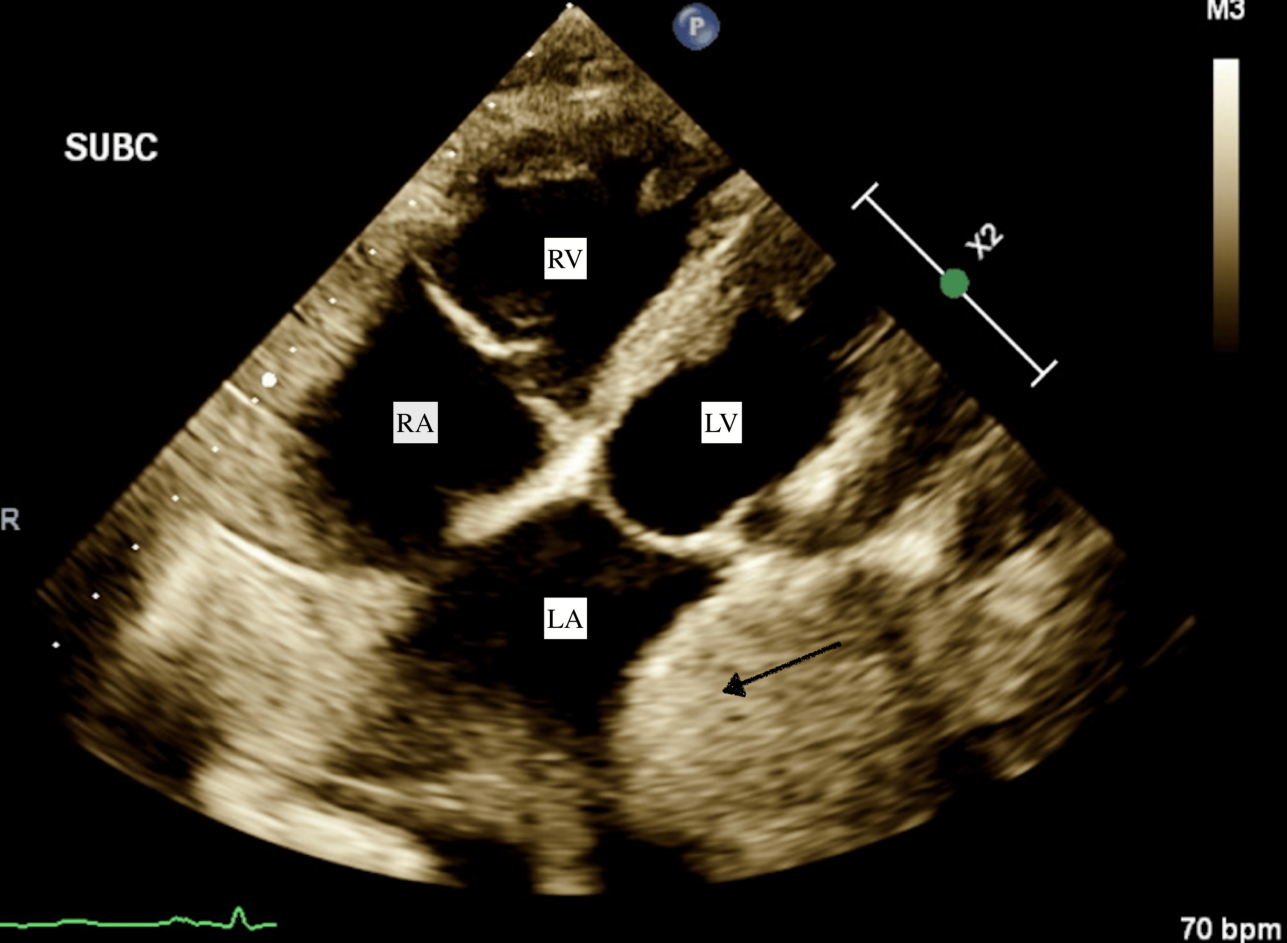
Echocardiographic subcostal 4 chamber view The arrow indicates an infiltrative lung mass compressing the left atrium (LA).

Further investigation with transthoracic echocardiogram (TTE) (Figures 2, 3) showed an extracardiac mass compressing and invading the lateral wall of the left atrium with extension to the basal portion of the left ventricle. Findings were concerning for extracardiac tumor infiltration. The ejection fraction was noted to be 60%. SVT was managed with diltiazem and metoprolol. The patient then developed respiratory distress with oxygen saturation dropping to 50%, leading to respiratory failure requiring intubation and mechanical ventilation. He subsequently went into cardiac arrest and was resuscitated. The patient was admitted and monitored continuously. He continued to develop recurrent episodes of SVT refractory to vagal maneuvers, requiring multiple chemical and electrical cardioversions.

**Figure 4:**
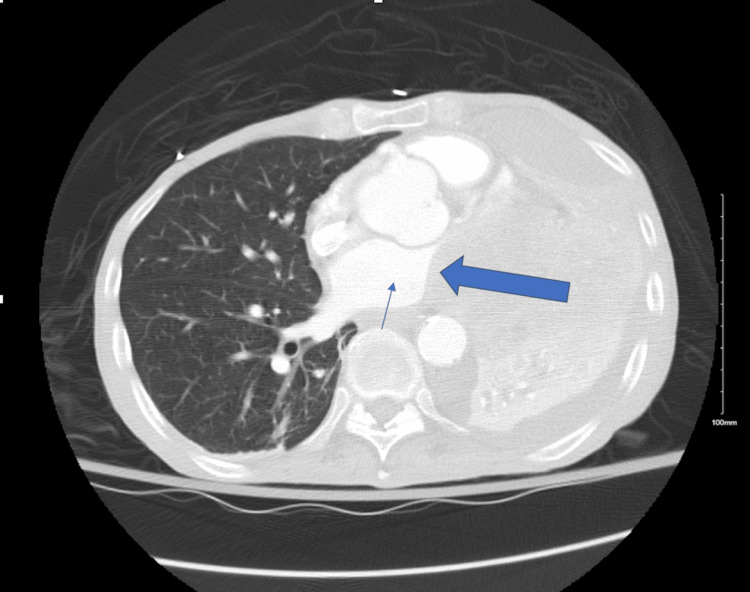
CT chest with contrast Shows mass compressing and invading the left atrium (big blue arrow) and left atrium (small blue arrow).

He was finally stabilized with amiodarone and metoprolol. Further investigation with computed tomography (CT) of the chest (Figure 4) revealed a necrotic mass in the left lower lobe infiltrating through the pericardium and occluding the left pulmonary veins as well as infiltration of the left atrium and left-sided coronary vessels. Endobronchial biopsy showed invasive moderately differentiated adenocarcinoma cytokeratin-7 (CK7), tumor protein p53 (p53), and proliferation marker Ki-67 (Ki-67) positive with a high proliferative index. The patient was evaluated by oncology, and the mass was deemed unresectable, with further workup considered unlikely to be beneficial. The patient was also not a candidate for systemic therapy. A plan was made for palliative radiation, and the patient was discharged home on metoprolol and amiodarone.

## Discussion

Cardiac tumors are rare, with a frequency of 0.0017%-0.33% [1]. Lung cancer accounts for about 36% of cardiac metastases and invasions, typically in advanced stages [2]. Pericardial metastasis, often clinically silent, may occur via lymphatic or hematogenous spread. One study found supraventricular tachycardia (SVT) in eight of 23 lung cancer patients with cardiac metastases, indicating a higher arrhythmia risk in this group [3]. Cardiac invasion and metastases as an initial cancer presentation are rare and can delay diagnosis and treatment.

Clinical presentation of intracardiac infiltration is variable, dependent on the location of the infiltration and compression of structures. Some clinical signs of cardiac involvement include cardiomegaly, development of congestive heart failure (CHF), or electrocardiographic changes [4]. Cardiac metastasis should be strongly suspected in a patient with known cancer who presents with sudden-onset unexplained tachycardia, arrhythmia, or CHF [5]. In high-risk individuals, maintaining a strong suspicion for cardiac involvement, conducting timely diagnostic evaluations, and initiating prompt treatment may significantly improve patient outcomes.

Atrial arrhythmias as the initial manifestation of a mediastinal tumor invading atrial structures are exceedingly rare, though a few cases have been documented in the literature. SVT or atrial tachyarrhythmias may occur when malignancies extend through the pulmonary veins into the left atrial myocardium [6]. One proposed mechanism is tumor infiltration of the myocardial sleeve of the pulmonary veins, creating zones of altered conduction that promote micro-reentrant circuits [6]. Additionally, mechanical stretching of the pulmonary veins and adjacent atrial myocardium may provoke focal atrial tachycardia [7]. Tumor-induced alterations in atrial conduction pathways may also lead to triggered automaticity [7]. Another contributing mechanism involves local invasion of the autonomic ganglionic plexuses at the pulmonary vein-left atrial junction, where disruption-along with mechanical stretch-can precipitate various forms of atrial tachyarrhythmia [8].

The evaluation of malignancy-related cardiac involvement typically begins with transthoracic echocardiography (TTE), which is the preferred initial imaging modality for assessing cardiac tumors and differentiating them from other cardiac conditions [9]. With advancements in ultrasound technology, transesophageal echocardiography (TEE) offers superior resolution, providing detailed visualization of the heart’s structures and pulmonary veins [10]. Further characterization of the mass is achieved through computed tomography (CT), which precisely defines the tumor’s location, its relationship to surrounding tissues, and helps identify other metastatic sites, serving as an effective initial screening tool for metastasis [11]. While definitive diagnosis of cardiac tumors requires pathological biopsy, its invasive nature often makes this approach unacceptable to many patients [12].

The prognosis of patients with malignant cardiac invasion largely depends on the extent of infiltration and the specific cardiac structures involved. Treatment approaches vary and are tailored to individual presentations. In one study, 38 patients with cardiac metastases from various malignancies were treated with radiation therapy, resulting in a 60% rate of clinical improvement over 12 to 36 months [5]. Additionally, a reported case of acute coronary syndrome (ACS) caused by coronary artery compression due to cervical cancer showed remarkable clinical improvement following one year of combined radiation and chemotherapy [13]. Ultimately, the treatment strategy is determined by the primary malignancy and the nature of the cardiac involvement, including whether surgical resection is feasible.

## Conclusions

This case underscores the importance of considering malignancy-related cardiac invasion in oncologic patients with new-onset arrhythmias and respiratory symptoms. Pulmonary adenocarcinoma can cause life-threatening SVT through direct intracardiac invasion. Early recognition, stabilization, and multidisciplinary management including tumor debulking and cardiology input are crucial for improving outcomes. There is a need for further case accumulation and research to better define optimal management and diagnostic pathways. 
